# Sulforaphane Ameliorates Metabolic Changes Associated With Status Epilepticus in Immature Rats

**DOI:** 10.3389/fncel.2022.855161

**Published:** 2022-03-15

**Authors:** Jan Daněk, Šárka Danačíková, David Kala, Jan Svoboda, Sonam Kapoor, Antonín Pošusta, Jaroslava Folbergrová, Kateřina Tauchmannová, Tomáš Mráček, Jakub Otáhal

**Affiliations:** ^1^Institute of Physiology, Czech Academy of Sciences, Prague, Czechia; ^2^Department of Pathophysiology, Second Faculty of Medicine, Charles University, Prague, Czechia

**Keywords:** status epilepticus, pilocarpine, immature rat, brain, μCT/PET, glucose metabolism, cerebral blood flow (CBF), sulforaphane

## Abstract

Status epilepticus (SE) is a common paediatric emergency with the highest incidence in the neonatal period and is a well-known epileptogenic insult. As previously established in various experimental and human studies, SE induces long-term alterations to brain metabolism, alterations that directly contribute to the development of epilepsy. To influence these changes, organic isothiocyanate compound sulforaphane (SFN) has been used in the present study for its known effect of enhancing antioxidative, cytoprotective, and metabolic cellular properties *via* the Nrf2 pathway. We have explored the effect of SFN in a model of acquired epilepsy induced by Li-Cl pilocarpine in immature rats (12 days old). Energy metabolites PCr, ATP, glucose, glycogen, and lactate were determined by enzymatic fluorimetric methods during the acute phase of SE. Protein expression was evaluated by Western blot (WB) analysis. Neuronal death was scored on the FluoroJadeB stained brain sections harvested 24 h after SE. To assess the effect of SFN on glucose metabolism we have performed a series of 18F-DG μCT/PET recordings 1 h, 1 day, and 3 weeks after the induction of SE. Responses of cerebral blood flow (CBF) to electrical stimulation and their influence by SFN were evaluated by laser Doppler flowmetry (LDF). We have demonstrated that the Nrf2 pathway is upregulated in the CNS of immature rats after SFN treatment. In the animals that had undergone SE, SFN was responsible for lowering glucose uptake in most regions 1 h after the induction of SE. Moreover, SFN partially reversed hypometabolism observed after 24 h and achieved full reversal at approximately 3 weeks after SE. Since no difference in cell death was observed in SFN treated group, these changes cannot be attributed to differences in neurodegeneration. SFN *per se* did not affect the glucose uptake at any given time point suggesting that SFN improves endogenous CNS ability to adapt to the epileptogenic insult. Furthermore, we had discovered that SFN improves blood flow and accelerates CBF response to electrical stimulation. Our findings suggest that SFN improves metabolic changes induced by SE which have been identified during epileptogenesis in various animal models of acquired epilepsy.

## Introduction

Epilepsy is the fourth most common neurological disease (Hirtz et al., 2007), seriously affecting the quality of life of the patients. Just in the United States, as of 2017, there were around 3.4 million epilepsy patients with 477,000 of them being child epilepsy patients (Zack and Kobau, 2017). Although most cases are treatable with antiepileptic drugs (AEDs), about a third of the epilepsy patients are presented with pharmacoresistant forms of epilepsy (Weaver and Pohlmann-Eden, 2013), with temporal lobe epilepsy (TLE) being the most common refractory subtype (Engel, 1996).

There is an ongoing search for new drugs that could diminish the number of refractory cases and bring relief to the patients (Weaver and Pohlmann-Eden, 2013). A substantial amount of research is aimed onto the process of how epilepsy develops over time (epileptogenesis) – i. e., changes developing after the initial insult [such as stroke, traumatic brain injury, status epilepticus (SE), and others] and leading to the occurrence of recurrent unprovoked seizures (Pitkänen et al., 2015; Broekaart et al., 2018). By targeting epileptogenesis, we should be able to prevent acquired epilepsies.

It has been widely established that epilepsy is associated with brain metabolic changes (Dubé et al., 2000, 2001; Lee et al., 2012; Bazzigaluppi et al., 2017; Malkov et al., 2018), for review see Otáhal et al. (2014) or Kovács et al. (2018). This process goes both ways – seizures cause metabolic alterations and metabolic alterations contribute to the occurrence of seizures.

Under physiological conditions, glucose presents the main energy source for the brain and unlike other tissues, the brain does not utilise other energy fuels such as fatty acids (Schönfeld and Reiser, 2013). During the seizures, demand for oxygen and glucose is increased, however, the production of energy is not sufficient (Bazzigaluppi et al., 2017). The reason behind this is a decrease in the activities of various enzymes involved in the tricarboxylic acid (TCA) cycle, such as malate dehydrogenase or aconitase (Bazzigaluppi et al., 2017). Moreover, maximal activities of another two TCA cycle participants, pyruvate dehydrogenase, and 2-oxoglutarate dehydrogenase complexes have been decreased (by 33 and 55%, respectively) in a murine hippocampus of adult mice subjected to Pilocarpine-induced SE (McDonald et al., 2017). On the contrary, the expression of enzymes involved in anaerobic glycolytic metabolism increases. A shift toward anaerobic metabolism is accompanied by increased lactate production, as has been observed in both human and experimental epilepsy. Reduced levels of oxygen during seizures induce the expression of hypoxia-inducible factors (HIFs) which further inhibit the TCA cycle and activate glycolysis (Bazzigaluppi et al., 2017). Mitochondria play an important role in the regulation of various cellular processes including the production of reactive oxygen species (ROS) (Martínez-Reyes and Chandel, 2020). Massive production of ROS during SE leads to alterations to cell components and organelles. Particularly, mitochondria seem to be highly sensitive to oxidative stress. Severe impairment of respiratory chain Complex I activity was detected in hippocampal CA3 neurons from patients with chronic TLE (Kunz et al., 2000), as well as in CA1 and CA3 hippocampal fields of rats after pilocarpine (Kudin et al., 2004) or kainic acid (Ryan et al., 2012) induced SE. The marked decrease of Complex I activity was also demonstrated in cerebral cortex mitochondria of immature rats after SE was induced by DL-Homocysteic acid (DL-HCA) (Folbergrová et al., 2007, 2010), lithium chloride (Li-Cl) pilocarpine, and kainate (Folbergrová et al., 2016, 2018). This decrease persisted during long periods of survival (up to 5 weeks), corresponding in these models to the development of spontaneous seizures. We have recently confirmed elevated ROS production during epileptogenesis in the Li-Cl pilocarpine model with significant elevations as long as 4 weeks after SE induced in the 12-days old rat pups (Folbergrová et al., 2018). Inhibition of the Complex I can form a vicious circle while it leads to enhanced production of ROS. The decrease was substantially reduced by treatment with selected free radical scavengers, namely by the superoxide dismutase (SOD) mimetics.

During acute seizures, epileptic focus dramatically increases the uptake of glucose, which is then visible on 18F-DG PET scans as a “hypermetabolic” region when compared to the background (Von Oertzen, 2018). We have shown *in vivo* massive depletion of glucose and energy reserves (glycogen and phosphocreatine) during the acute phase of pilocarpine SE in immature rats (Folbergrová et al., 2007, 2016). On the other hand, interictal hypometabolism has been demonstrated in a wide range of epileptic syndromes including TLE, generalized childhood absence epilepsy, and SE (Kovács et al., 2018). Although seizure-induced cell loss and sclerotic modification of the tissue could partially explain the hypometabolic state, there is also evidence for dysfunction of the neurometabolic coupling (Kann et al., 2005) and oxidative damage of respiratory enzymes, reviewed in Folbergrová and Kunz (2012). Recent findings of a group of Zilberter (Malkov et al., 2018, 2019) showed direct interaction of ROS with glycolysis (ROS decrease glucose utilisation). It is worth hypothesizing that hypometabolism could be at least partially caused by the increased production of ROS. Our experiment focuses on the reversal of all these changes which should manifest as brain hypermetabolism observed in the acute phase of epileptogenesis – immediately post insult (hours) (da Silva Fernandes et al., 1999; Pereira de Vasconcelos et al., 2002), followed by hypometabolism in the latent epileptogenesis phase (days-weeks in animals, even months in human patients) (Dubé et al., 2001; Knowlton et al., 2001; Sarikaya, 2015; McDonald et al., 2017).

The isothiocyanate sulforaphane (SFN), isolated from extracts of broccoli, has been shown as a potent naturally occurring activator of nuclear factor erythroid two-related factor 2 (Nrf2), the major regulator of the cellular response to oxidative stress (Denzer et al., 2016). Mazzuferi et al. (2013) reported that overexpression of Nrf2 in adult mice 2 weeks after pilocarpine-induced SE, provided an enormous protective effect, evident as the reduced number and frequency of spontaneous recurrent seizures, marked reduction in the number of activated microglia and preservation of hippocampal neurons. Other authors reported that in adult rats an activation of the Nrf2 pathway by sulforaphane leads to the suppression of amygdala kindling progression, amelioration of oxidative stress, and cognitive impairments induced by seizures (Wang et al., 2014). Pauletti et al. (2019) have demonstrated that in adult animals undergoing epileptogenesis a transient 2-week treatment with N-acetylcysteine and SFN significantly delayed the onset of epilepsy, blocked disease progression, and reduced the frequency of spontaneous recurrent seizures (Pauletti et al., 2019).

In our study, we have selected SFN to evaluate its effect on metabolic changes associated with SE in immature rats. The aim of our study was to answer important and clinically relevant questions: First, does SFN cause Nrf2/ARE pathway activation in immature rats? Second, if it does, does the activation of Nrf2 with SFN pretreatment modify glucose uptake and reverse metabolic changes induced by SE in immature rats? Third, does SFN modify cerebral blood flow (CBF) regulation in immature rats?

## Materials and Methods

### Animals

In total, 65 immature 10 days old male Wistar rats from the local colony (Institute of Physiology CAS) were used for these experiments. The protocol of the experiments (number 50/2017) was approved by the Animal Care and Use Committee of the Institute of Physiology, Czech Academy of Sciences, in agreement with the Animal Protection Law of the Czech Republic, which is fully compatible with the guidelines of the European Community Council directives 2010/63/EU. All efforts were made to minimize animal suffering and to reduce the number of animals used.

### Induction of Status Epilepticus

Lithium chloride (Li-Cl) pilocarpine model of SE for immature rats was used. The model is well established in our department (Folbergrová et al., 2018). Firstly, 12-day-old rats (PD12) were chosen for SE induction because of the level of brain maturation which is comparable to the early postnatal period in human infants (Dobbing, 1970). To induce SE, 24 h before the induction of SE (on a postnatal day 11; PD11), rat pups were given *i.p.* injection of Li-Cl (127 mg/kg, Sigma-Aldrich) dissolved in distilled water. On PD12 SE was induced by *i.p.* injection of pilocarpine (Pilo; 35 mg/kg; Sigma-Aldrich) dissolved in distilled water. Control animals received corresponding volumes of the appropriate vehicles. In all Li-Cl Pilocarpine treated animals, latency to the onset of the first occurrence of clonic movements of one or both forelimbs, intensity, and frequency of clonic seizures were continuously observed. The detailed character of seizures was described in our previous work (Folbergrová et al., 2016). In the acute and subacute group, the animals were returned approximately after 2 h to their mothers for selected periods of survival.

### Experimental Groups and Sulforaphane Treatment

The animals were divided into four groups, with their pretreatment described in Table 1. In two experimental groups, SE was induced by Li-Cl Pilocarpine and two groups served as controls. Groups received either SFN or an appropriate vehicle. The rat pups in the SFN treatment groups were receiving *i.p.* injections of SFN (APExBIO, United States) dissolved in dimethyl sulfoxide + PBS (final concentration of DMSO ∼0.5%) on day 10 and day 11 (PD10 and PD11, respectively). The treatment dose was set to 5 mg/kg based on previously published data (Alfieri et al., 2013) and our pilot experiments.

**TABLE 1:** Design of experimental setup.

Treatment day	SE groups		Control groups	
	Pilo	Pilo + SFN	Saline	Saline + SFN
PD10	DMSO + PBS	SFN (5 mg/kg)	Saline	SFN (5 mg/kg)
PD11	DMSO + PBS + Li-Cl	SFN (5 mg/kg) + Li-Cl	Saline + Li-Cl	SFN (5 mg/kg) + Li-Cl
PD12	Pilo	Pilo	Saline	Saline

To determine glucose uptake, we have used FDG uptake measured by PET at three different time stages: acute (1-h post SE induction, PD12), subacute (24 h post SE induction, PD13), and latent (3 weeks post-SE induction, PD34). Due to demanding experimental procedures, we have used an independent subset of animals (*n* = 11) for acute experiments while another subset of animals (*n* = 9) was used in both the subacute and latent stage allowing us to assess FDG uptake in the same animals.

### Energy Metabolites

To evaluate levels of energy metabolites PCr, ATP, glucose, glycogen, and lactate, enzymatic fluorimetric methods according to Lowry and Passoneau (1972) have been employed. In total, 10 animals have been used for these measurements. Briefly, the heads of the pups were frozen when falling directly into liquid nitrogen after decapitation approximately 30 min from the beginning of SE. Frozen brains were dissected out at −22°C and samples of the cerebral cortex (approximately 25 mg) were extracted at −30°C with HCl/methanol and subsequently at 0°C with perchloric acid, as described in more detail previously (Folbergrová et al., 2007, 2016). Levels of energy metabolites were determined by microfluorimetry (Lowry and Passoneau, 1972).

### Protein Electrophoresis and Western Blotting

To find out whether SFN affects its established pathways (Innamorato et al., 2008) and other genes/proteins associated with metabolism, Western blot (WB) was performed on saline (*n* = 4 animals) and saline + SFN groups (*n* = 4 animals).

Tissue lysates of the hippocampus (10%, w/v) were prepared at 4°C in RIPA medium (150 mM NaCl, 1% Nonidet NP-40, 1% sodium deoxycholate, 50 mM Tris-Cl, pH 8) containing protease inhibitor cocktail (1:500, Sigma P8340) and phosphatase inhibitors (1:200, Sigma P5726) by grinding on Retsch MM400 grinder (Retsch Gmbh, Haan, Germany) (zirconium oxide grinding balls at 30 Hz for 3 min). Lysates were spined down (10,000 g for 10 min), and resulting supernatants were used for subsequent analyses.

Sodium dodecyl-sulphate polyacrylamide gel electrophoresis (SDS PAGE) was performed by established protocol (Nuskova et al., 2019). After electrotransfer, membranes were blocked in Protein-free blocking buffer (37572, Pierce). Primary and secondary antibodies used for immunodetection are detailed in Supplementary Table 1. Detection was performed using the fluorescence scanner Odyssey (LI-COR Biosciences), and signals were quantified by ImageLab software version 6 (Bio-Rad).

Poly(rC)-binding protein 1 (PCBP1) was used as a housekeeping protein (Lee et al., 2016).

### Animal Weight Analysis

Since the Li-Cl pilocarpine model is associated with weight loss we aimed to assess the effect of SFN on animal weight. The animals’ weight (*n* = 16) was determined using standard laboratory weight every morning (except for the days when the PET analysis was scheduled).

### Neuronal Death Analysis

To evaluate neuronal death after SE, we have scored neuronal death using FluoroJade B immunohistochemistry (IHC) in our animals (*n* = 8) 1 day after SE induction (on PD13). The animals have undergone fixation procedure as follows: after urethane anaesthesia, animals were transcardially perfused with buffered saline followed by 4% fresh paraformaldehyde. After 3 h of post-fixation, the brains were moved to sucrose (10, 20, and 30%) for cryoprotection. After freezing, 50-μm-thin coronal sections were cut and stained with FluoroJade B (Histochem, United States) as previously used and described in detail by our group (Folbergrová et al., 2008, 2016). We set to compare neurodegeneration between the Pilo and Pilo + SFN groups in mediodorsal thalamic nuclei (MDN), piriform cortex (PC), and hippocampal regions CA1, CA3, and DG. Slices were analysed using the upright fluorescence microscope (Olympus BX53, Japan) under 10× magnification. Three (MDN, within −2 to −2.75 mm from bregma) or five (CA1, CA3, DG, within −3 to 4.5 mm from bregma; PC, within 0 to −1.5 mm from bregma according to Paxinos and Watson, 1998) slices per region of interest (ROI) were evaluated and a semiquantitative scale was used to assess the neuronal death – score 0: < 7 stained neurons; score 1: 7–15 neurons score 2: 16–25 neurons; score 3: 26–40 neurons, score 4: > 40 neurons per ROI and slice. Maximal reached score per ROI and brain was used for further analysis.

### 18F-DG PET Scanning and Data Analysis

To assess changes in glucose metabolism, a small animal PET scanning device (Albira Si, Bruker, United States) with a spatial resolution of up to 0.7 mm was used (Albira, Bruker). Fluorodeoxyglucose (18F-DG; ÚJV, Czech Republic) has been used in our experiments. Our team has expertise with 18F-DG use in small animals (Svoboda et al., 2019). Animals were intravenously injected (jugular vein) with the selected dose of 18F-DG (10 – 15 MBq for PD12 and PD13 animals and 20–22 MBq for PD34 animals) dissolved in 0.2 or 0.4 (PD34) ml of saline, while shortly anaesthetized by isoflurane (ISF). After the 18F-DG dose injection, we had waited for approximately 45 min for the 18F-DG uptake into the brain to occur (Sarikaya, 2015). Finally, animals were again anaesthetized by ISF and gently placed into the observation chamber. Due to the technical limitations, the collected data are limited to the animal’s head in each case. Each animal’s PET scan took 45 min. The PMOD software version 3.6.1 (PMOD technologies LLC, Zurich, Switzerland) was used for the offline analysis of data. Co-registration of the PET scan with Schiffer’s MRI rat brain atlas (Schiffer et al., 2006) implementing the PET scan into Paxinos coordinates was performed by trained specialists.

For measuring the changes in glucose uptake, we decided to use a standardised uptake value (SUV) as calculated by PMOD. The following formula is used by PMOD software to calculate SUV:


(1) $$\text{SUV}=\frac{\text{A}\times \text{W}\times \text{V}}{\text{D}}\left[g\right]$$

With the variables being: A – Activity concentration in the image (kBq/cc); D – Applied dose (kBq) at the time the image is corrected to; W – animal weight (g); V – ROI volume (cc).

The calculation returns the ratio of injected dose (ID) per the given ROI, multiplied by the animal weight.

Then for the analysis, we have determined ROIs based on their availability in the Schiffer’s MRI rat brain atlas and the overlap with the regions affected the most in the Li-Cl Pilocarpine model (limbic regions and others) (Druga et al., 2003; Guo et al., 2009). According to the distinction of the PMOD software between the left and right hemisphere for any given ROI, there are 2 data points for a single ROI.

Additionally, macro-ROIs comprising of previously selected ROIs were established. The macro-ROIs are as follows: hippocampus (Hip), cortex (Cx), thalamus (Thal), and Midbrain (Mid). Hip macro-ROI consists of the anterodorsal hippocampus (AD Hip) and posterior hippocampus (P Hip), totalling 4 data points per animal. Cx macro-ROI consists of the motor cortex (MCx), the somatosensory cortex (SCx), visual cortex (VCx), prefrontal cortex (PCx), and entorhinal cortex (ECx), totalling 10 data points per animal. Thal macro-ROI consists of Thalamus (Thal) and inferior colliculus (IC), totalling 4 data points per animal, and finally, Midbrain macro-ROI consists of Midbrain (Midbrain) and ventral tegmental area (VTA), totalling 4 data points per animal.

We have used the average value for the voxel of the given ROI during the 45 min measurement period (which was normalized to 1 cc). Since the volumes of certain ROIs are very small, we decided to use this value for a better overview of the measured data and a more concise reading experience. Therefore, the SUV ROIs data in this article are normalized to 1 cc of the tissue (even for macro-ROI where the selected ROIs are simply pooled together).

### Cerebral Blood Flow Recordings and Analysis

Upon discovering an article showing the effect of SFN on blood flow (Parfenova et al., 2020) in guinea pigs we have decided to determine whether upon electrical stimulation SFN-pretreated immature rats show different blood flow responses when compared to blank rat pups. Blood flow changes were assessed via Laser Doppler flowmetry (LDF) after electrical stimulation of the mirror cortical region (transcallosal stimulation). Detected CBF changes thus represent the response of the neurovascular unit to synaptic neurotransmitter release. The surgical procedure was as follows: Animals (in total 13 animals, 5 in Saline + SFN and 8 in Saline group) were anaesthetised using *i.p.* injection of urethane (1.1 g/kg, 20% solution) and body temperature was kept constant at 37 ± 0.1°C using a heating pad with close loop control during the entire experiment. An incision at the level of sagittal suture was performed to access the skull. Two stimulation epidural electrodes were placed above the left sensorimotor cortex (craniotomy coordinates P12: 2–3 mm lateral, 1 mm anterior, and 1 mm posterior), and LDF sensor (Perimed, Sweden) was positioned on the exposed contralateral (right) parietal bone to detect CBF responses from the contralateral cortex (coordinates P12: 2–3 mm lateral, 0 mm posterior bregma). While parietal bone is thin and transparent at this age, we were able to detect signals through the intact skull.

The stimulation protocol was as follows: stimulation pulses were biphasic (100 μs, 5 mA) throughout the course of the experiments. In the first experiment, animals were receiving short-lasting (5 s) electrical stimulation of increasing frequency (5, 10, 15, and 20 Hz) to mimic different levels of neuronal activity to construct a dose (input-output) curve. Each stimulation was followed by at least 5 min lasting break for a full recovery. The LDF baseline was calculated as an average of the 5 s period before the stimulation onset (for each frequency and animal separately). After recording responses for input-output curve, the second experiment began – the very same rat pups were subjected to stimulation protocol which mimics SE like activity (10 s lasting 20 Hz stimulation, 10 s break, another 10 s lasting 20 Hz stimulation – 5 of such stimulations in 90 s was performed). The LDF baseline was calculated as an average of the 5 s period before the SE-like stimulation onset (before the first of the five stimulations, for each frequency and animal separately). All the data are displayed as % of the baseline.

Due to the nature of neurovascular coupling in immature rodents (Zehendner et al., 2013), first, we observed a drop in CBF followed by blood flow recovery/overshoot (Figure 7A). Therefore, for the stimulation of increased frequency, parameters assessed (during the 30 s time window starting with the stimulation onset) were as follows – area under the curve (enabling us to determine increase/decrease of blood flow), local minimum (largest observable blood flow decrease; L_min_), local maximum (largest observable blood flow increase; L_max_), amplitude (L_max_ – L_min_), average change (average difference from the baseline throughout the entire recording, set in absolute value), times to reach L_min_ and L_max_ (times were calculated from the onset of the stimulation; T_Lmin_ and T_Lmax_), time to cover amplitude (T_Lmax_ – T_Lmin_) and finally amplitude velocity ((L_max_ – L_min_)/(T_Lmax_ – T_Lmin_)). The very same parameters were used for the analysis of the SE-like stimulation (Figure 7A) with the only difference that the time window was 20 s (from initiation of one stimulation to initiation of another with 20 s time window after the onset of final stimulation). Only the parameters in which SFN was a significant factor on the two-way ANOVA (with factors of SFN and selected parameter) are shown in this article.

### Statistical Analysis

As we were primarily interested in the effect of SFN on the reversal of metabolic changes associated with SE, Kruskal–Wallis test was used, comparing all the four groups together in the single given time point. The Kruskal-Wallis test was then followed by *post-hoc* Dunn’s multiple comparisons (MCs) test.

Where applicable (CBF and WB experiment), two-way ANOVA between both vehicle/blank groups with factors of SFN and one variable factor was performed to discover whether SFN has an effect across the variable factor.

All the statistical analyses mentioned above were performed using GraphPad Prism software version 9.3.1. (GraphPad Software, United States).

## Results

All animals treated with Li-Cl pilocarpine developed SE exhibiting the same patterns as previously described (Folbergrová et al., 2016). Approximately 10 min after the application of pilocarpine animals became restless, with occasional scratching and trembling, followed by the occurrence of clonic movements of one and later both forelimbs, sometimes accompanied by rearing. The intensity and frequency of clonic seizures gradually increased, in many animals complemented by falling on either side. SFN pretreatment did not alter latency, character, duration, or severity of seizures as well as mortality (Supplementary Figure 1).

### Sulforaphane Causes Upregulation of Nrf2 and Nrf2-ARE Regulated Proteins in PD12 Rats

**FIGURE 1:**
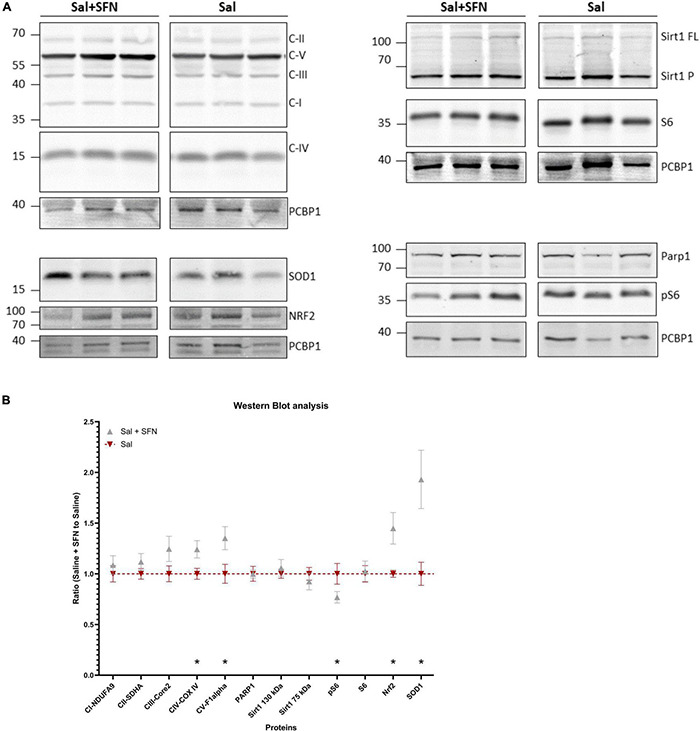
Western blotting (WB) analysis normalized to the housekeeping protein (PCBP1). The data shown are from animals that differ only by sulforaphane (SFN) pretreatment, no status epilepticus (SE) was initiated. The SFN group was further normalized to the Control animals – the ratio of SFN-treated animals to Control animals is shown. **(A)** Representative gels from the same animals (Sirt1FL = Sirt1 130 kDa; Sirt1P = Sirt1 75 kDa). **(B)** WB signal quantification. We observe significantly increased expression of Nrf2 and its direct (SOD1) and indirect (CIV and CV) targets, along with pS6 decrease (two-way ANOVA with factors of Protein and SFN comparing Saline vs. Saline + SFN group: Interaction *P* < 0.05, Protein *P* < 0.05, SFN *P* < 0.001; Multiple uncorrected unpaired *t*-tests were run to compare expression differences between the groups at each protein. All values are reported as mean ± SEM (*P < 0.05).

First, we aimed to establish whether SFN causes upregulation of Nrf2 *via* its well-established pathway (Alfieri et al., 2013); according to our knowledge, *in vivo* experiments involving the action of SFN in immature rats had not been performed. The Western blot to measure protein levels was used. Furthermore, proteins such as mitochondrial complexes (Complex I-V) and proteins involved in NAD^+^ consumption (Sirt1, PARP1) were also measured. Representative gels (always for three animals per group) are shown in Figure 1A and their subsequent quantification in Figure 1B. SFN treatment results in a significant increase of Nrf2 as well as in Nrf2-ARE regulated SOD1. Similarly, we observed a significant increase for representative subunits of mitochondrial respiratory chain complexes IV and V, an indirect target for Nrf2 (Gureev et al., 2019). We also observed a decreased phosphorylation of S6 protein (pS6 marker, Figure 1B), an established downstream target of mTOR (Leontieva et al., 2012). We conclude that SFN/Keap1/Nrf2 pathway is active under our experimental conditions in the immature brain. For the original WB gels see Supplementary Dataset 1.

### Sulforaphane Does Not Reverse Weight Loss Caused by Li-Cl Pilo Induced Status Epilepticus

**FIGURE 2:**
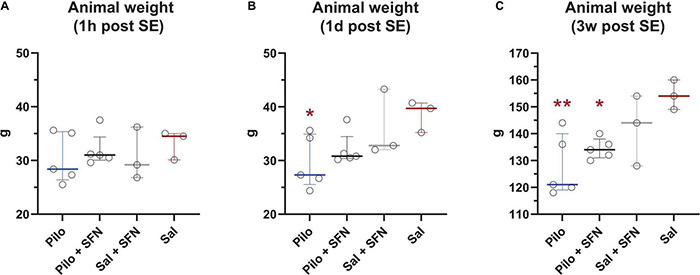
Effect of SFN on animal weight. 1 h post SE, we observe no statistically significant weight changes between the groups **(A)**. We observed reduced weight in SE groups 1 day **(B)** and 3 weeks after SE **(C)**. SFN has no effect on weight gain neither in the Saline nor in the SE groups (The Kruskal–Wallis test was followed by uncorrected Dunn’s test comparing all other groups to the Saline group, *P < 0.05 vs. Sal group). All values are reported as median ± interquartile range. **P < 0.01.

Since the Li-Cl Pilocarpine model is associated with weight loss (Jiang et al., 2013; Imran et al., 2015), we initially focussed on its potential mitigation by SFN. The PD12 animal weights (Figure 2A) were very similar within the groups. However, the day after (Figure 2B) the Pilo group weight was significantly lower (*P* < 0.05) while the Pilo + SFN group also exhibited weight loss, albeit not significant when compared to the control group. Decreased body weight was present in SE groups also at 3 weeks interval independently of the SFN treatment (Figure 2C) as we observe a significant difference between Saline and both Pilo groups (*P* < 0.05).

### Sulforaphane Does Not Have Any Impact on the Mild Neuronal Death Observed 24 h After Status Epilepticus

**FIGURE 3:**
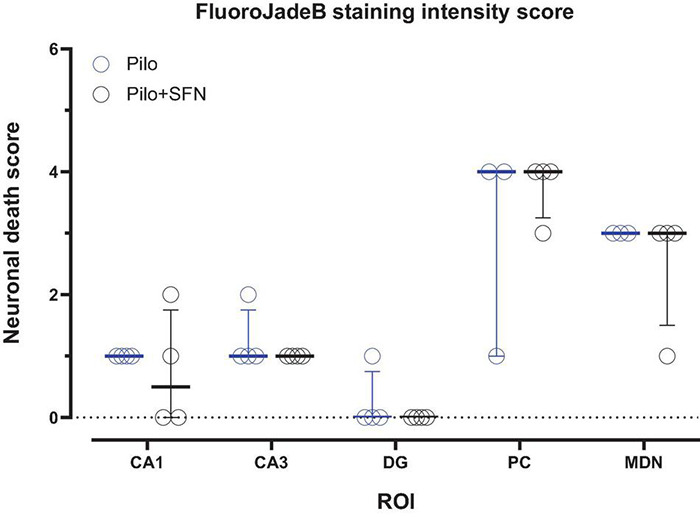
SFN pretreatment had no impact on the neuronal death measured 24 h after the induction of SE (multiple unpaired Mann–Whitney tests comparing Pilo and Pilo + SFN group). All values are reported as median ± interquartile range.

To see whether our 18F-DG-PET results might have been affected by diminished radionucleotide uptake as a consequence of neurodegeneration, we have performed an evaluation of the FluoroJadeB positive cells in five areas (CA1, CA3, DG, PC, and MDN) in which mild neuronal damage is typically present in this model of the SE in the immature rats (da Silva Fernandes et al., 1999; Druga et al., 2003; Leroy et al., 2003; Folbergrová et al., 2016). No statistically significant changes in neurodegeneration evaluated in defined ROIs have been found between Pilo and Pilo + SFN groups 24 h after SE (Figure 3), the time period normally associated with the most profound neuronal damage in the Li-Cl Pilo induced SE model (Schauwecker, 2012).

#### Sulforaphane Pretreatment Has a Beneficial Effect on Glucose Uptake After Status Epilepticus

**FIGURE 4:**
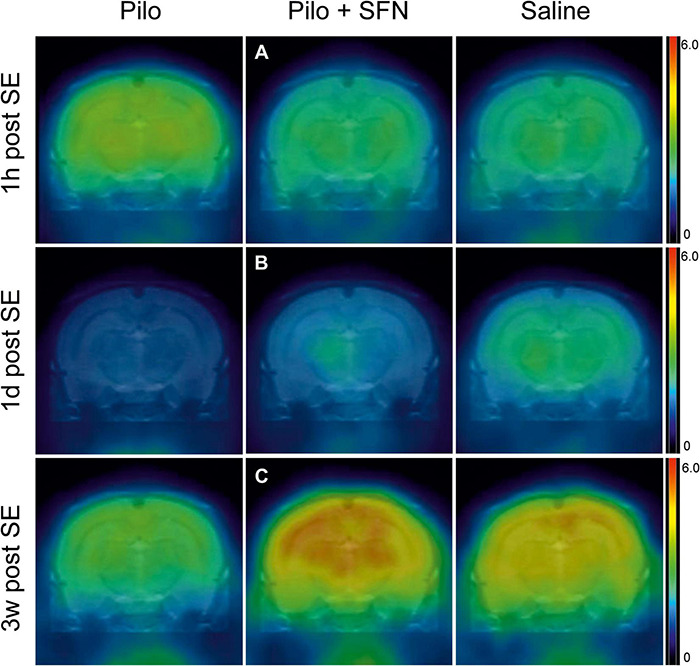
Representative 18F-DG-PET measurements from all the three-time periods, coronal section images taken on the level of the hippocampus and the thalamus. SFN pretreatment prevents hypermetabolism 1 h post-SE **(A)**, partially restores hypometabolism 1-day post SE **(B)**, and seems to lead to glucose uptake above both control and Pilo group levels 3 weeks post SE **(C)**. All the representative samples are reported in the SUV units (g).

**FIGURE 5:**
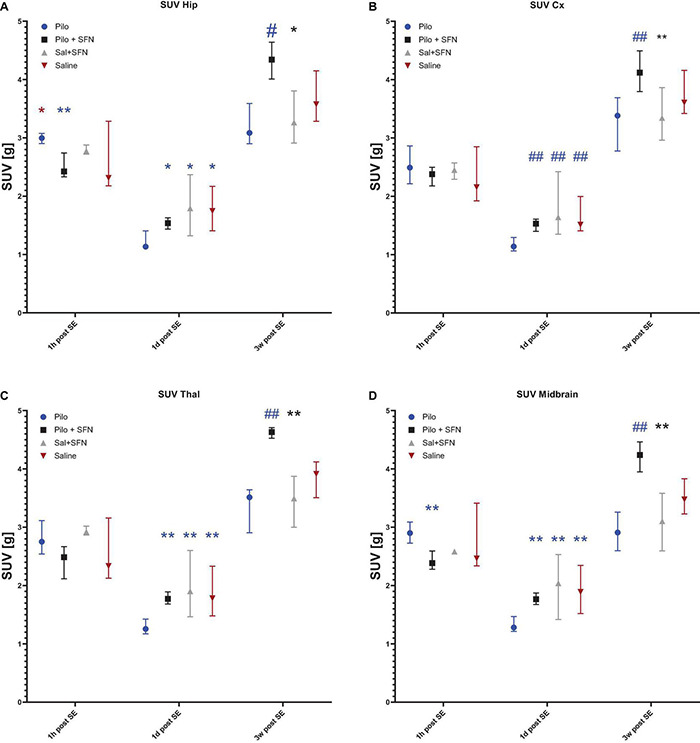
SFN pretreatment reverses changes in glucose uptake and enhances recovery after SE in all the macro-ROIs. In the hip **(A)** and midbrain **(D)**, macro-ROIs differences between the groups appeared in the earliest time point (Kruskal–Wallis test *P* < 0.05) while SFN attenuated glucose uptake increase 1 h post SE. Additionally, SFN influenced glucose uptake in all the ROIs **(A–D)** both 1 day and 3 weeks post SE (Kruskal–Wallis test *P* < 0.05 in all the macro-ROIs in both time periods). Symbols in the graph come from *post-hoc* Dunn’s multiple comparisons test that was conducted if the differences on the Kruskal-Wallis test had shown significant. The colour of the symbol denotes the group the comparison was made to, and the location of the symbol denotes which was the other group. All values are reported as median ± interquartile range (*P < 0.05, **P < 0.01, ^#^*P* < 0.001, ^##^*P* < 0.0001).

To find out whether there is an effect of SFN on glucose metabolism we performed a series of PET scans after the application of FDG at three time points after the SE (Figure 4). Although SFN does not possess the ability to alter glucose uptake under physiological conditions (as shown later) we have observed significant beneficial effects of SFN pretreatment in the SE group which is in agreement with previously published data (Carrasco-Pozo et al., 2015; Denzer et al., 2016). The effect was evaluated at each time point separately (due to hypothesized different effects of SFN in the acute vs. subacute and latent period) and the results are given individually in the following sections (Figures 4, 5).

#### Sulforaphane Pretreatment Normalizes Glucose Hypermetabolism 1 h After Status Epilepticus in the Hippocampus and Midbrain

In total, 11 animals were selected for this time period (Figure 5A). SE led to an apparent increase of FDG uptake, as a result of increased brain energy demand (18F-DG was applied 1 h after the start of SE and measured 45 min later; see Figure 4A for illustration). We have found significant differences in two macro-regions of interest (Kruskal–Wallis test *P* < 0.05), namely Hippocampus (Figure 5A) and Midbrain (Figure 5D). We see that in these macro-ROIs, SFN pretreatment reduced glucose hypermetabolism when comparing Pilo and Pilo + SFN groups. Despite the fact that SFN had no effect on the SE characteristics (Supplementary Figure 1) we already observe the metabolism-modifying effect in the acute period 1 h after SE cessation. Furthermore, the Hip macro-ROI SFN pre-treatment reversed a significant difference between Pilo and Saline groups.

#### Sulforaphane Pretreatment Reverses Hypometabolism 1 day and 3 Weeks After Status Epilepticus

One day after SE induction, the same protocol for 18F-DG-PET as described above was used. In total nine animals were selected for this period (Figure 5B). SE had rendered the brain hypometabolic when compared to the control (Saline) group (see Figure 4B for illustration). During this time period, SFN had been shown to not only exert its effect (Kruskal–Wallis test: *P* < 0.05) in all the macro-ROIs (Figures 5A-D), but most importantly, the Pilo + SFN group had shown a significant increase in the SUV value in all the macro-ROIs (Figures 5A–D) which was matching our hypothesis. SUV values of the Pilo group were always lower than SUV values of all the other groups.

The same nine animals as in the previous period were used for the final time period. Three weeks after the end of the acute phase we have found that Li-Cl Pilo still renders the brain hypometabolic when compared to the control (Saline) group (see Figure 4C for illustration). SFN pretreatment had sustained its beneficial effect when it comes to the reversal of the decreased glucose uptake observed in the Pilo group (by approximately 25%) (see Figures 5A–D).

#### Sulforaphane Pretreatment Does Not Impact Levels of Metabolites Assessed During the Acute Phase of Status Epilepticus

**FIGURE 6:**
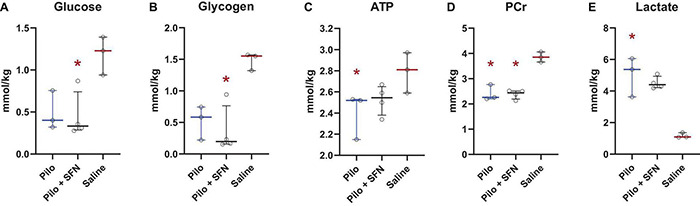
SFN pretreatment does not lead to any significant changes in the brain metabolites, namely Glucose **(A)**, Glycogen **(B)**, ATP **(C)**, PCr **(D)** and Lactate **(E)**, determined in Pilo groups when assessed 30 min after the onset of SE. The colour of the significance symbol (*) denotes the group the comparison was made to, and the location of the symbol denotes which was the other group. The Kruskal–Wallis test was followed by *post-hoc* uncorrected Dunn’s test. All values are reported as median ± interquartile range (*p < 0.05).

Upon measuring the concentrations of glucose (Figure 6A) and various other metabolites, namely glycogen, ATP, phosphocreatine (PCr), and lactate (Figures 6B–E) in the cerebral cortex 30 min after SE onset we see a significant decrease in concentrations of glucose (Figure 6A), glycogen (Figure 6B) and PCr (Figure 6D) in the Pilo + SFN group and a significant increase in lactate levels (Figure 6E) and decrease in ATP concentration (Figure 6C) in the Pilo group when compared to Saline. However, we have not detected any statistically significant change in the levels of these metabolites between the Pilo and Pilo + SFN groups. We conclude that during the acute phase of SE, all the available metabolites which may serve as an energy source are depleted and SFN has no impact on this phenomenon, at least within the first 30 min of SE.

#### Sulforaphane Increases Cerebral Blood Flow and Accelerates Its Response to Stimulation

**FIGURE 7:**
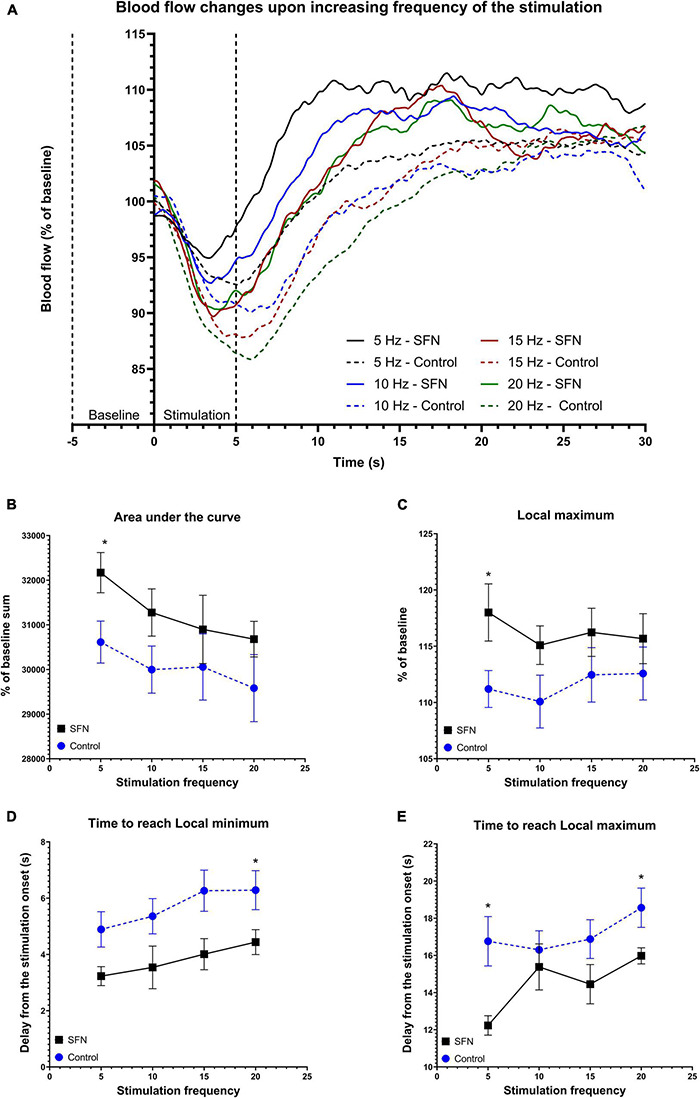
SFN pretreatment improved blood flow and accelerated response to the electrical stimulation of increasing frequency. Baseline was calculated as average of values from 5 s before the stimulation to its onset. The average value at any given time (smoothed line) for each frequency and group is shown panel **(A)**. Dotted lines denote the beginning of baseline measurement and end of stimulation. Parameters that had shown SFN as a significant factor on two-way ANOVA are shown panels **(B–E)**. We observe increased AUC **(B)** suggesting increased total blood flow during the 30 s period, increased L_max_ **(C)**, and quicker response as shown when comparing both time to reach L_min_ **(D)** and L_max_ **(E)**. Multiple unpaired *t*-tests were run to compare values at each frequency, significant results are denoted by an asterisk. All values **(B–E)** are reported as mean ± SEM (*P < 0.05).

**FIGURE 8:**
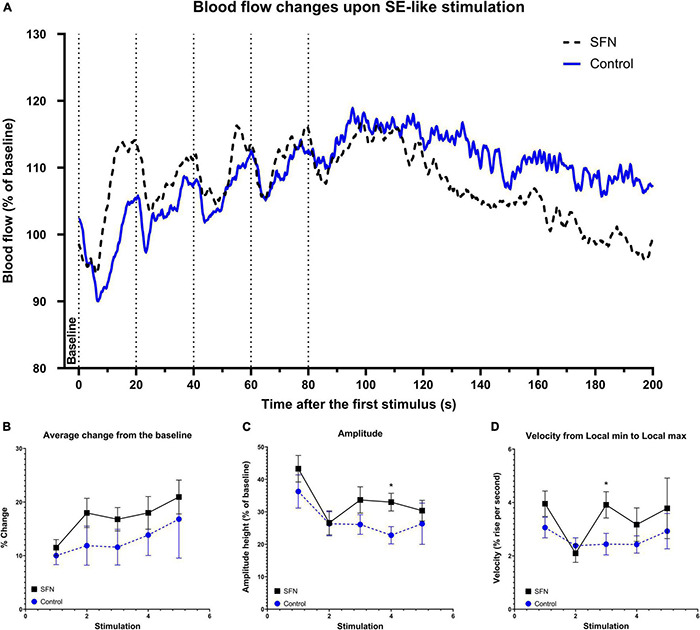
SFN pretreatment improved blood flow response to SE-like stimulation. Dotted lines **(A)** denote the beginning of each 20 Hz stimulation lasting 10 s. These 5 rapid consecutive stimulations with no given time to full recovery were used to simulate a SE-like impact on the blood flow. Baseline was calculated as average of values from 5 s before the first stimulation to its onset. The average trace of the group at any given time (smoothed) for each frequency and group is shown panel **(A)**. Parameters that had shown SFN as a significant factor (two-way ANOVA) are shown panels **(B–D)**. We observe the increased average change from the baseline **(B)** increased amplitude **(C)** and increased velocity to cover the amplitude **(D)**. Multiple unpaired *t*-tests were run to compare values at each stimulus, significant results are denoted by an asterisk. All values **(B–D)** are reported as mean ± SEM (*P < 0.05).

To verify whether SFN has effects on CBF as reported recently (Parfenova et al., 2020) as CBF is also tied with glucose uptake (Huisman et al., 2012), LDF recording was performed in our experiments. Transcallosal stimulation with linearly increased frequency (Figure 7A) and SE-like stimulation (Figure 8A) was used. The parameters employed are described in the section “Materials and Methods.” SFN was shown to significantly influence CBF responses as elicited by increasing stimulation frequencies, specifically in the area under the curve (AUC; Figure 7B; suggesting increased blood flow), L_max_ (Figure 7C), and T_Lmin_ and T_Lmax_ (Figures 7D–E; suggesting a faster response of CBF to the stimulation). When the same animals had been given SE-like stimulation, SFN significantly altered the Average change from the baseline (Figure 8B; suggesting increased responsivity to the stimulation), Amplitude (Figure 8C; suggesting increased range of response), and finally Amplitude velocity (Figure 8D; suggesting a faster restoration of the blood flow to the desired range when compared to the control).

These results suggest that SFN has beneficial CBF modifying properties which might contribute to the better supply of substrates and wash-out of metabolites during the acute phase of SE.

## Discussion

The main objective of this study was to test whether SFN, a molecule showing promising clinical potential in the adult brain (Denzer et al., 2016; Zhang, 2017; Pauletti et al., 2019) can reverse metabolic changes induced by strong epileptogenic insult, namely Li-Cl pilocarpine SE induced in the immature rat pups. As epilepsy is tightly connected with lasting alterations in metabolism in epileptogenic tissue (Otáhal et al., 2014; Kovács et al., 2018), prevention of these changes would allow curbing the process of development of acquired epilepsy (patients after hypoxic-ischemic insults, TBI, tumours, stroke, with neurodegenerative diseases, multiple sclerosis, and various other conditions).

Indeed, disruption of the energy metabolism has been shown in both experimental and human epilepsies (Otáhal et al., 2014; Kovács et al., 2018). Various mechanisms of how metabolic changes directly contribute to epileptogenesis have been discovered and solid evidence exists on seizure-induced damage to the brain metabolism in both adult and immature brain (Folbergrová and Kunz, 2012; Otáhal et al., 2014; Kovács et al., 2018).

Epileptogenic insults such as SE result in massive production of ROS in acute as well as in the latent period after the event in both adult and immature brains (Waldbaum and Patel, 2010; Folbergrová et al., 2012, 2016, 2018; Otáhal et al., 2014). Markers of oxidative stress have been found also in human TLE tissue and interestingly also in dysplastic tissue in surgery material from focal cortical dysplasia patients (Arena et al., 2019). It has been shown by Kann et al. that the human hippocampus from TLE patients possesses alterations of mitochondrial metabolism (Kann et al., 2005). In addition to observations showing the causal role of ROS in this phenomenon (Kovács et al., 2002), ROS have been shown to alter glycolysis during experimentally induced seizures *in vitro* recently (Malkov et al., 2018) and interact with seizure initiation (Malkov et al., 2019). Moreover, the same group observed that chronic inhibition of glycolysis with 2-deoxyglucose in rats led to the initiation of paroxysmal brain activity resembling epileptogenesis, and some treated rats developed tonic-clonic seizures (Samokhina et al., 2017). Despite the traditional concept of epileptogenesis increasing body of evidence indicates that disturbances especially in brain energy metabolism contribute to an increased propensity to generate seizures and thus to epileptogenesis.

Our strategy to prevent the above-mentioned metabolic changes in the Li-Cl pilocarpine model was to deliver SFN before induction of SE. SFN comes from cruciferous vegetables, its concentration is high especially in young broccoli sprouts, and is a potent Nrf2 transcription factor activator. Nrf2 represents the master regulator of cellular redox homeostasis and is upregulated in the hippocampus resections from human TLE patients (Mazzuferi et al., 2013). Under the physiological conditions, it is a short-lived protein that is a target for its negative regulator Kelch-like-ECH-associated protein 1 (Keap1). Keap1 protein uses a cyclic mechanism to target Nrf2 for ubiquitination and proteasomal degradation. SFN distorts this process by binding to cysteine residues of Keap1, thereby inactivating Keap1, which leads to Nrf2 accumulation and its subsequent translocation into the nucleus where it binds to antioxidant-response elements (ARE) and initiates transcription of various genes with antioxidant and anti-inflammatory functions, proteasomal subunits and metabolic enzymes (Kwak et al., 2003; Zhang, 2017). Production of GSH is directly regulated by Nrf2 that has also an impact on NADPH-generating enzymes. Other enzymes affected by Nrf2 include SOD1, HO-1, GPx1, malic enzyme 3, and thioredoxins enzyme class essential for the reduction of oxidized thiols. On top of the previous findings, Nrf2 deficient systems such as knock-out cells or animals are much more sensitive to the toxicity of oxidants (Dinkova-Kostova and Abramov, 2015). Interestingly, the mechanism of SFN action is not limited to Nrf2 activation. It has also been shown to inhibit the mTOR axis (Wiczk et al., 2012; Zhang et al., 2019), a pathway also very important in both animal and human epileptogenesis (Wong, 2013; Crino, 2015; Citraro et al., 2016).

Sulforaphane (SFN) has been shown to have a beneficial effect in experimental models of epilepsy in adult animals. Previous investigation of SFN in epilepsy *in vivo* conducted on adult rats treated with SFN and N-acetylcysteine (NCA, GSH precursor) has shown delays in the epilepsy onset and decreased frequency of spontaneously recurrent seizures at five months after initial SE. Average seizure duration, along with the epilepsy incidence between treatment and vehicle group has not changed (Pauletti et al., 2019). Another set of *in vivo* experiments conducted on CD31 adult mice has shown that daily injections of SFN for 5 days elevated the seizure thresholds to 6 Hz stimulation and fluorothyl-, but not pentylenetetrazole-induced tonic seizures and exhibited neuroprotective effects after SE induced by pilocarpine (Carrasco-Pozo et al., 2015). This raises the crucial question of whether the protective effect of SFN could potentially be due to an anticonvulsant effect and thus less severe initial insult. Our data however do not reveal an effect of SFN on character, severity, latency, or duration of seizures during Li-Cl pilocarpine induced SE in immature rats as revealed by EEG analysis (Supplementary Figure 1). In addition, similar findings i.e., lack of anticonvulsant effect is what we have observed previously in the same model of SE in immature rats with another antioxidative drug resveratrol which possesses both a ROS scavenging and Nrf2 activating effect (Folbergrová et al., 2021). The differences between our observations and those of Carrasco-Pozo et al. (2015) might be due to different models involved (pilocarpine vs. Li-Cl pilocarpine), differences between immature and adult brain and most likely due to different parameters evaluated (EEG analysis vs. percentage of developed SE). Thus, our findings are compatible with the statement that the SFN does not alter the severity nor character of Li-Cl pilocarpine SE i.e., initial insult in immature rats.

To elucidate an effect of SFN in the immature brain during experimentally induced SE we performed a series of experiments in 12-days old rat pups during and after Li-Cl Pilo SE. To measure metabolic alterations, we employed 18F-DG-PET, a tool commonly used to monitor glucose consumption *in vivo* (Yao et al., 2012).

Furthermore, 1 h after SE onset, we observed a mild increase in glucose uptake with SFN counterbalancing this effect. Hypermetabolism occurring 1 h after initiation of Li-Cl Pilo SE has also been previously observed (mostly in the regions related to SE propagation) in 10-days old Sprague–Dawley immature rats as assessed using a local cerebral metabolic rate of glucose (LCMR_glc_) measured by [^14^C]2-DG (da Silva Fernandes et al., 1999; Dubé et al., 2000). In these rats, 1 h after SE glucose uptake increases ranged from 63 to 625% compared to the control in almost all cortices and forebrain areas with > 450% increases in the PC, amygdala, CA1 of the hippocampus, and dentate gyrus (DG) hilus. However, no statistically significant changes had been observed in cerebral and arterial glucose concentrations in these rats (da Silva Fernandes et al., 1999). Data we collected are also in correlation with the increase in mRNA expression of GLUT1 (main astrocytic glucose transporter securing glucose supply from blood as astrocytic endfeet are part of the blood-brain barrier) in the hippocampus, entorhinal and piriform cortices, and basolateral amygdala 1-h after Li-Cl Pilo SE in 10-days old Sprague–Dawley immature rats. Furthermore, the mRNA of GLUT3 (main neuronal glucose transporter) is elevated in the hippocampus, PC, basolateral amygdala, and mediodorsal thalamus. This elevation was much higher (maximum around 325% of control for GLUT3 and 200% for GLUT1) than in 21-days old and adult rats because of low basal levels of GLUT1 and GLUT3 in the immature brain (Leroy et al., 2011).

In the subacute phase, 1 day post SE, we observed hypometabolism in the Pilo group, which was partially, but significantly, reversed by pretreatment with SFN. Hypometabolism starting 1 day after SE has been observed by other authors as well (Dubé et al., 2001; Kovács et al., 2018). Nevertheless, it is worth highlighting that this phenomenon (hypermetabolism – hypometabolism switch) is generally more profound in adult animals (Pereira de Vasconcelos et al., 2002). Additionally, it might be tempting to consider neuronal death responsible for the hypometabolism one-day post SE, however that is not the case for immature animals (used in our experiment), where we see damage restricted only to certain regions (Druga et al., 2003).

Observed hypometabolism 1 day after SE is also in accordance with studies measuring N-acetyl aspartate (NAA) levels (NAA is synthesised only by neuronal mitochondria and is used as a putative marker of oxidative metabolism) which have revealed that its levels decrease in the interictal phase in mTLE in various patient populations and mTLE animal models (Lee et al., 2012; Bazzigaluppi et al., 2017). On top of that, GLUT3 mRNA 1-day after Li-Cl Pilo induced SE is decreased compared to the control, although not significantly, with GLUT1 still elevated, although not as much as during the first hour after SE (Leroy et al., 2011). We assume that the above-mentioned post-SE transcriptional changes have been the reason why we have not observed the recovery to the levels of the control group 1 day after SE – the effect of the SFN has been (partially) counterbalanced by the post-SE shift from the TCA cycle to the glycolysis (Bazzigaluppi et al., 2017). Moreover, in a previous experiment, Keap-1 knockdown cells in cortical or midbrain neurons and glial mixed cultures have not changed expression of Complexes I, II, and IV, suggesting that Nrf2 stimulation cannot counterbalance TCA enzymes decrease post SE – although the mitochondrial oxidative phosphorylation is more efficient in Nrf2-stimulated cells (Holmström et al., 2013). Yet in our experimental setting, we have observed Complex IV and ATP synthase (Complex V) to increase in the control animals treated with SFN.

Our last time point of μCT/PET assessment marked as latent phase (3 weeks after SE onset) occurs when glucose and oxidative phosphorylation have already been established as a main brain energy source and acute neuroinflammation should have faded. Although hypometabolism is still observed in the Pilo group, we have observed supranormal (above Saline group) SUV levels in the Pilo + SFN group in all ROIs. These findings indicate the long-lasting effect of SFN pretreatment on energy metabolism changes observed after SE induced at postnatal day 12. These observations could be likely attributed to various factors; however, only limited data are available in the literature preventing to fully understanding and explaining the mechanisms involved. Several tempting hypotheses can be mentioned, although their involvement is highly speculative. The first of them is the ability of the SFN (Nrf2) to permanently alter GLUTs expression after early postnatal exposure. The second hypothesis could be that SFN has a long-term impact on mitochondrial functions. It has been shown, that RTA-408, another Nrf2 stimulator, increases mitochondrial functions (assessed by GSH and ATP levels) in the hippocampus and cortex to supranormal levels (Shekh-Ahmad et al., 2018), possibly increasing requirements of mitochondria for substrates (Aguiar et al., 2012), leading to transcriptional changes resulting in higher uptake of glucose. The most intriguing hypothesis in terms of the development of epilepsy is the possible interaction of Nrf2 with GABA_A_ inhibition. Several experiments suggest that Nrf2 activation speeds up NADH regeneration in a seizure-like environment (Greco et al., 2011; Dinkova-Kostova and Abramov, 2015; Kubo et al., 2017). The presence of the NAD^+^ cofactor (along with G3P, ATP, and Mg^2+^) is vital for the autophosphorylation of the α_1_ subunit of GABA_A_R (Laschet et al., 2004). Exogenous phosphorylation is delivered by a kinase directly bound to the receptor, glyceraldehyde-3-phosphate dehydrogenase (GAPDH), which is also a key glycolytic enzyme. Together with phosphoglycerate kinase (PGK) also localised at the neuronal membrane, they allow the production of ATP which is then directly used to autophosphorylate GADPH. The pGADPH then transfers the phosphate to the α_1_ subunit of the GABA_A_R, maintaining its physiological functions. *In vitro* whole-cell patch-clamp recordings experiments have shown that if this endogenous phosphorylation is prevented, the rundown of GABA_A_ currents is accelerated (Laschet et al., 2004). Experiments working with human epileptic surgically removed tissue have indeed found reduced endogenous phosphorylation of GABA_A_R that has not resulted from a decrease of the α_1_ subunits (Pumain et al., 2008). We speculate this endogenous phosphorylation of GABA_A_R is restored in our model meaning there is an increased substrate pool (NAD^+^, G3K, ATP). As G3K is a product (downstream) of glucose, increased glucose uptake could lead to a restoral of the GABA_A_R function in epileptic tissue. This is even strengthened by the fact that the supranormal glucose uptake is observed only in the Pilo + SFN group suggesting the phenomenon resulted from the combination of the SE insult and SFN pretreatment. However, further work is desired to elucidate relevant pathophysiological mechanisms.

The beneficial effects of SFN likely extend beyond antioxidative action. It has recently been shown that SFN exerts CBF modifying properties in guinea pigs (Parfenova et al., 2020) *via* increased activation of enzymes responsible for endogenous H_2_S production followed by activation of K_ATP_ and BK on the smooth muscle (Patel et al., 2018). It is a question of what mechanism caused the vasodilatory effect of SFN in our experiments. It should be emphasised that very low concentrations of SFN have been used by Parfenova et al. and according to these authors SFN was acting *via* a non-genomic mechanism (Parfenova et al., 2020). We have employed much higher concentrations of SFN, leading to Nrf2 activation. It is thus not clear whether the non-genomic or Nrf2 dependent mechanism is responsible for the effect observed in our conditions. Future experiments are needed for clarification of this issue.

Upon the analysis of the LDF data, we have noticed that there is a peculiar biphasic response present (in the adult animals we have observed only an increase of the blood flow without the initial decrease – data not shown). Zehendner et al. (2013) have described similar phenomena in the barrel cortex of PD7 mice (Zehendner et al., 2013). The initial decrease of CBF was explained by different neuronal properties at that age as assessed *via* local field potentials (LFPs) and multi-unit activity (MUA). In the PD7 animals, MUA was significantly delayed when compared to LDF, presumably because the white matter is not yet fully developed so conduction velocity should be slower (Wang et al., 2008). Furthermore, in the PD7 mice, there has been an initial MUA peak followed by an increased activity for a couple of seconds (MUA was more stable in PD30 animals) then followed by a decreased incidence of spontaneous MUAs post stimuli in PD7 animals, suggesting overshoot of the neuronal activity to the extent there is a “multi-unit activity fatigue” triggering the CBF decrease. To support this hypothesis, there is also a negative correlation between CBF and MUA over time (unlike a positive correlation in PD30 animals). Paired pulse experiments have further revealed decreased excitability upon repetitive stimulation in PD7 animals (Zehendner et al., 2013). The phenomena observed in this experiment in PD7 mice well corresponds to our observations concerning CBF in PD12 rats.

In our model, we have shown that SFN increases CBF upon electrical stimulation, an effect that is even more pronounced in adult animals (manuscript in preparation). SFN further quickens the time to reach the initial CBF drop (local minimum) before reaching maximum blood flow (which is increased in SFN pretreated animals and SFN also aids to reach local maximum quicker as well). In the context of SE-mimicking stimulation, we have observed that SFN increased the amplitude of the biphasic response and also the speed to cover the amplitude. These results suggest SFN has beneficial CBF modifying properties and could perhaps also interact with postictal and interictal hypoperfusion (Farrell et al., 2016) as both of these phenomena are often observed in epilepsy.

## Conclusion

We have shown the beneficial impact of short SFN pretreatment on the selected metabolic parameters in CNS as measured after Li-Cl pilocarpine-induced SE in immature rats. The present data indicate that in 12-days old rat pups SFN exerts its effect *via* the same pathways as in adult rodents, namely, primarily Nrf2/ARE pathway. SFN pretreatment not only reverses hypermetabolism in the acute phase but also improves hypometabolism starting 1 day after SE (subacute phase) and lasting at least up to 22 days after onset of SE (latent phase), as observed in our experiments. SFN also exerts a beneficial effect on blood flow. Our findings suggest that SFN improves metabolic changes induced by SE which have been identified during epileptogenesis in various animal models of acquired epilepsy.

## Data Availability Statement

The raw data supporting the conclusions of this article will be made available by the authors, without undue reservation.

## Ethics Statement

The animal study was reviewed and approved by the Animal Care and Use Committee of the Institute of Physiology, Czech Academy of Sciences.

## Author Contributions

JD: μCT/PET analysis, performed the CBF recordings, the overall data analysis, wrote the draft of the manuscript. ŠD: data analysis, writing, and editing of the manuscript. DK: μCT/PET data processing. JS and SK: μCT/PET experiments. AP: data processing and interpretation. JF: study design, measurements of energy metabolites, and manuscript editing. KT: performed the Western blot analyses. TM: conducted the Western blot analysis and interpretation and manuscript editing. JO: principal investigator, study design, supervision of all experiments and data analysis, performed part of μCT/PET and CBF experiments, final editing, and proofreading. All authors contributed to the article and approved the submitted version.

## Conflict of Interest

The authors declare that the research was conducted in the absence of any commercial or financial relationships that could be construed as a potential conflict of interest.

## Publisher’s Note

All claims expressed in this article are solely those of the authors and do not necessarily represent those of their affiliated organizations, or those of the publisher, the editors and the reviewers. Any product that may be evaluated in this article, or claim that may be made by its manufacturer, is not guaranteed or endorsed by the publisher.

## Supplementary Material

Supplementary Figure 1 **(A)** EEG activity during Li-Cl Pilo status epilepticus has been determined as the number of epileptic spikes in 60 s bins. Time zero is the time of pilocarpine application. Red line, convulsant agent alone (*n* = 6); Blue line, convulsant agent plus SFN (*n* = 5). **(B)** A total number of detected epileptic spikes during 90 min of SE duration was not influenced by SFN suggesting no anticonvulsant effect (*P* = 0.67). Latency to start of behavioural SE after pilocarpine application also did not differ between the groups *P* = 0.76. EEG was recorded from silver epidural electrodes placed above the sensorimotor cortex. Epileptic spikes were detected using Spike2 software (CED, United Kingdom) and analysed in Matlab (Mathworks, Nattick, MA, United States).
