# Supplementary figures and images for: Sulforaphane Ameliorates Metabolic Changes Associated With Status Epilepticus in Immature Rats
Source: Front Cell Neurosci. 2022 Mar 15;16:855161. doi: 10.3389/fncel.2022.855161 (PMC8965559; doi:10.3389/fncel.2022.855161)

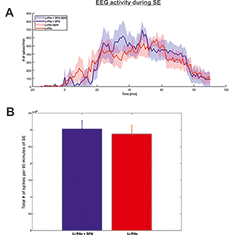

Supplement: Supplementary Figure 1 — (A) EEG activity during Li-Cl Pilo status epilepticus has been determined as the number of epileptic spikes in 60 s bins. Time zero is the time of pilocarpine application. Red line, convulsant agent alone (n = 6); Blue line, convulsant agent plus SFN (n = 5). (B) A total number of detected epileptic spikes during 90 min of SE duration was not influenced by SFN suggesting no anticonvulsant effect (P = 0.67). Latency to start of behavioural SE after pilocarpine application also did not differ between the groups P = 0.76. EEG was recorded from silver epidural electrodes placed above the sensorimotor cortex. Epileptic spikes were detected using Spike2 software (CED, United Kingdom) and analysed in Matlab (Mathworks, Nattick, MA, United States). [file Image_1.tif]
