# Supplementary material for: Sulforaphane Ameliorates Metabolic Changes Associated With Status Epilepticus in Immature Rats
Source: Front Cell Neurosci. 2022 Mar 15;16:855161. doi: 10.3389/fncel.2022.855161 (PMC8965559; doi:10.3389/fncel.2022.855161)
Supplement: Supplementary file 2 [file Table_1.docx]

Supplementary Table 1:

| **Primary antibodies** |  |  |  |
| --- | --- | --- | --- |
| **Target** | **producer** | **Cat. No.** | **dilution** |
| MS603 BN OXPHOS Kit | Abcam | ab110412 | 1:250 |
| ribosomal protein S6 | SCBT | sc-74576 | 1:100 |
| phospho-ribosomal protein S6 (Ser 235/236) | SCBT | sc-293144 | 1:200 |
| SIRT1 | SCBT | sc-74504 | 1:100 |
| PARP-1 | SCBT | sc-74470 | 1:100 |
| Poly(rC)-binding protein 1 (PCBP1 or hnRNP E1) | Cell Signalling | 8534S | 1:1000 |
| Nrf2 | Abcam | ab89443 | 1:500 |
| CuZnSOD (SOD1) | AbFrontier | LF-PA0013 | 1:1000 |
|  |  |  |  |
| **Secondary antibodies** |  |  |  |
| **target** | **producer** | **Cat. No.** | **dilution** |
| Alexa Fluor 680 Donkey anti-Mouse IgG (H+L) | Life Technologies | A10038 | 1:3000 |
| Alexa Fluor 680 Donkey anti-Rabbit IgG (H+L) | Life Technologies | A10043 | 1:3000 |
| IRDye 800CW Donkey anti-Mouse IgG (H+L) | LI-COR | 926-32212 | 1:15000 |
| IRDye 800CW Donkey anti-Rabbit IgG (H+L) | LI-COR | 926-32213 | 1:15000 |
