# Supplementary material for: Sulforaphane Ameliorates Metabolic Changes Associated With Status Epilepticus in Immature Rats
Source: Front Cell Neurosci. 2022 Mar 15;16:855161. doi: 10.3389/fncel.2022.855161 (PMC8965559; doi:10.3389/fncel.2022.855161)

Daněk et al. Sulforaphane ameliorates metabolic changes associated with status epilepticus in immature rats

Originals of WB gels

MS603 – OXPHOS coctail (for CI, CII, CIII, CIV, CV)

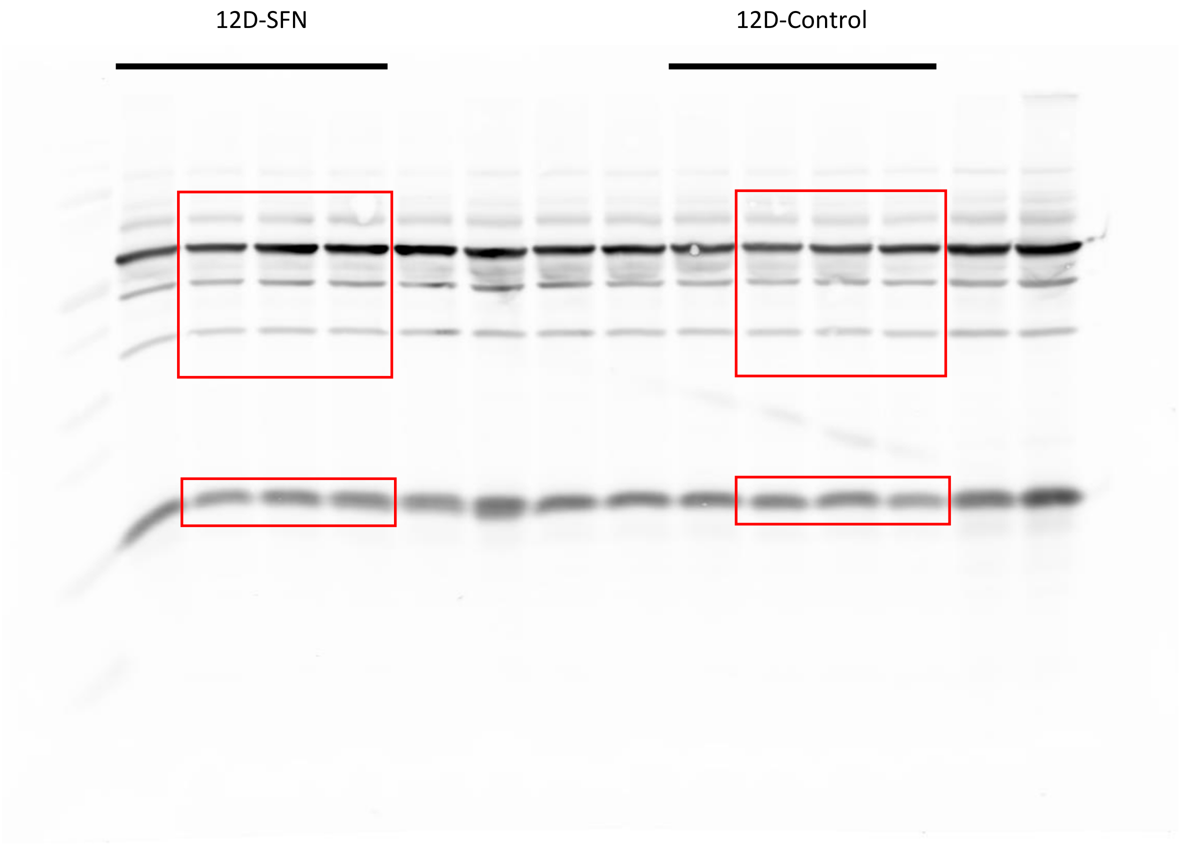

Pcbp1 for MS603 signal

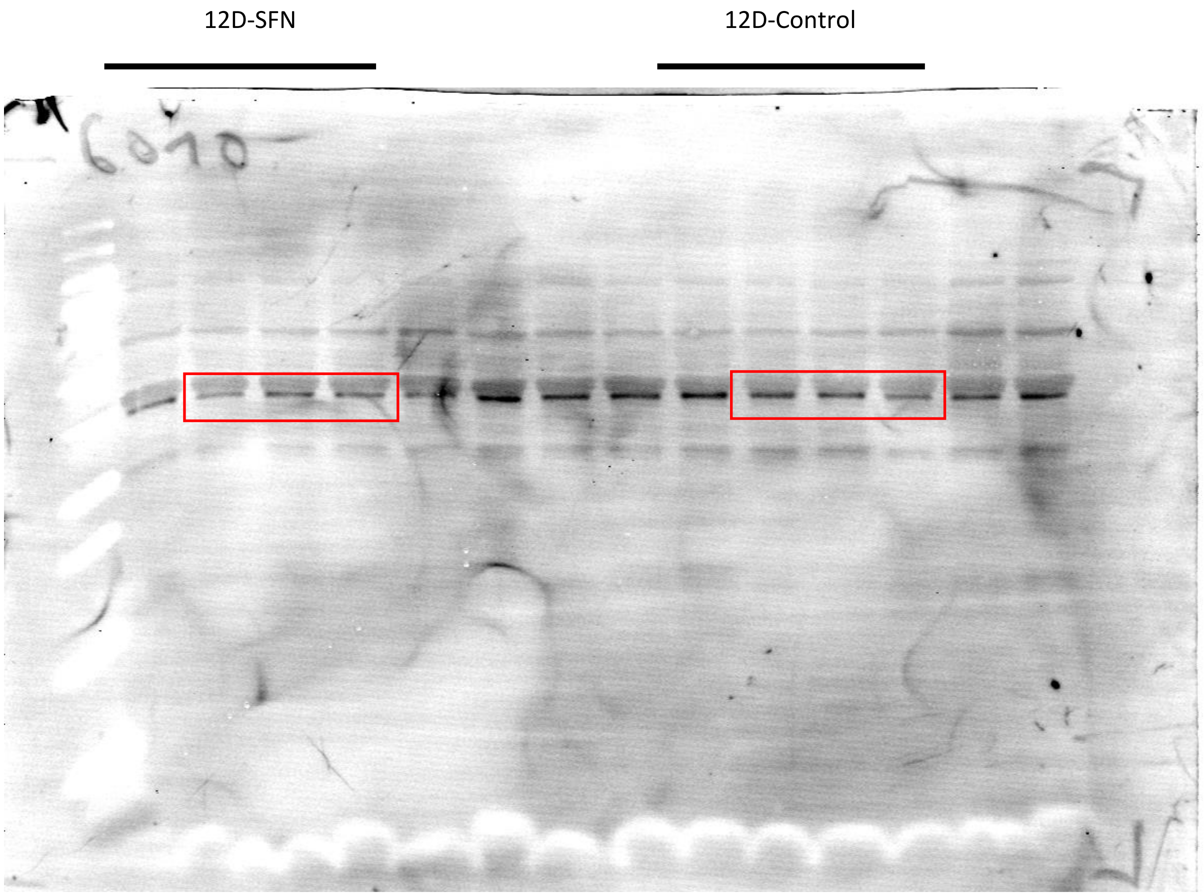

Nrf2

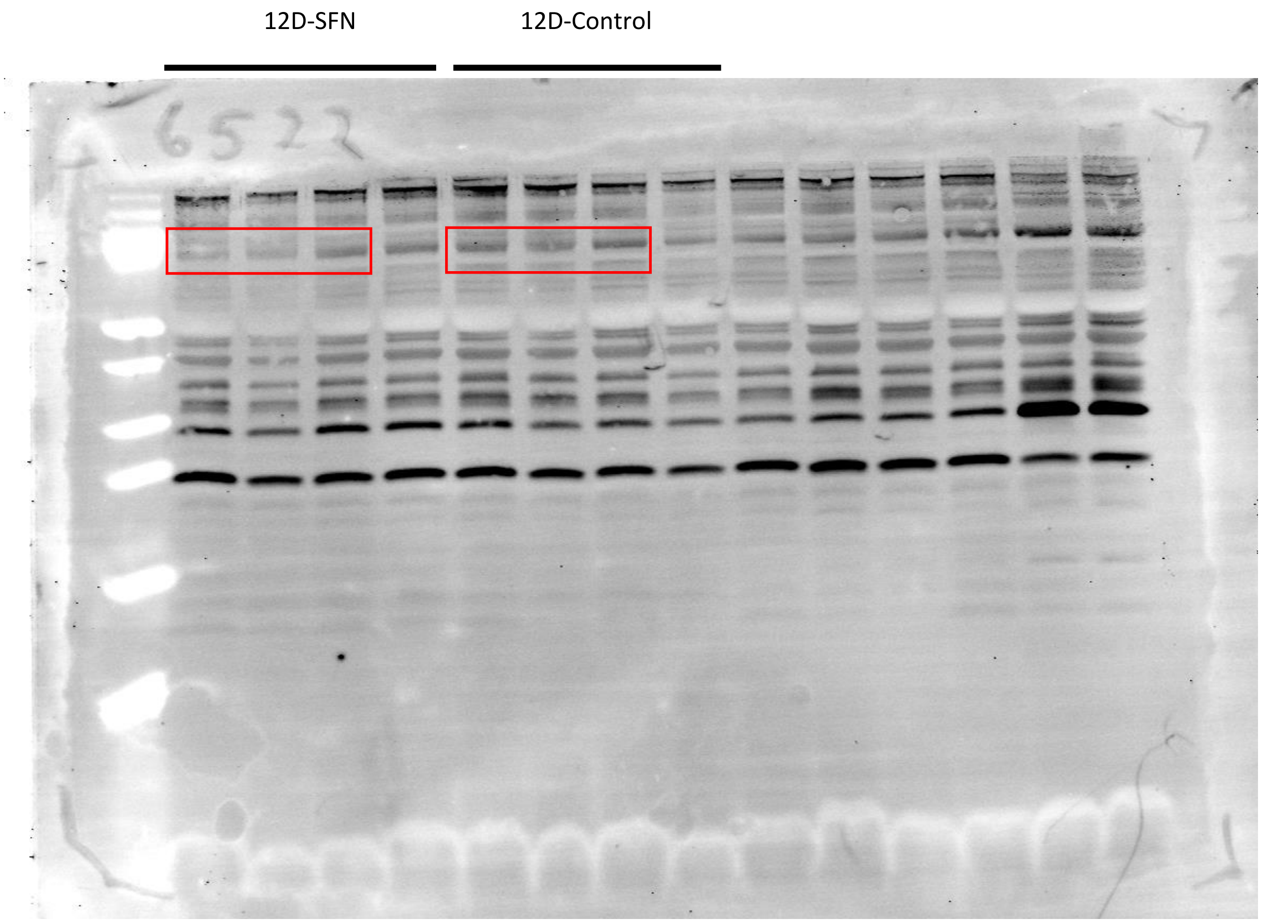

Sod1

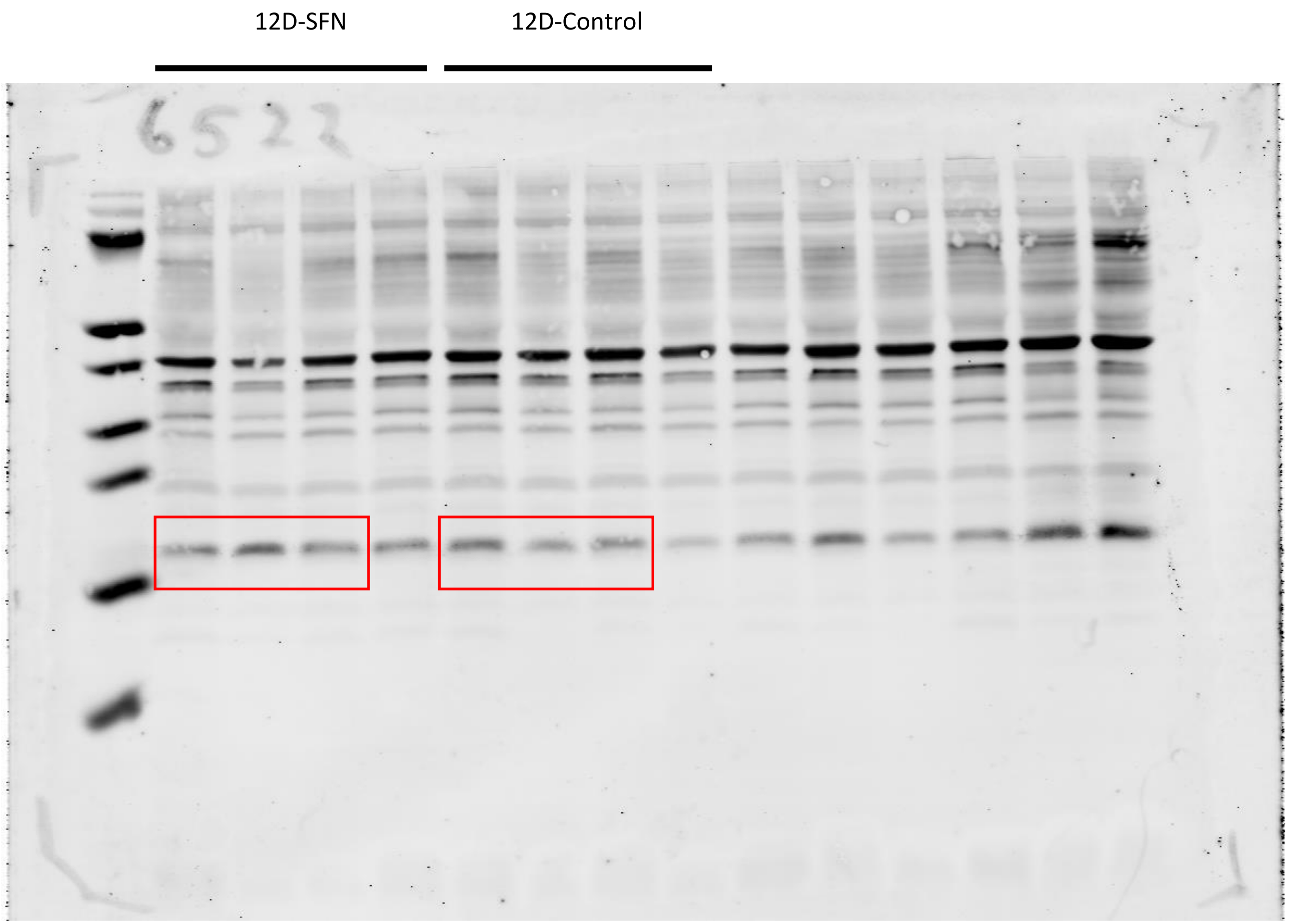

Pcbp1 – over Nrf2, for Nrf2 and SOD1 signal

12D-SFN 12D-Control

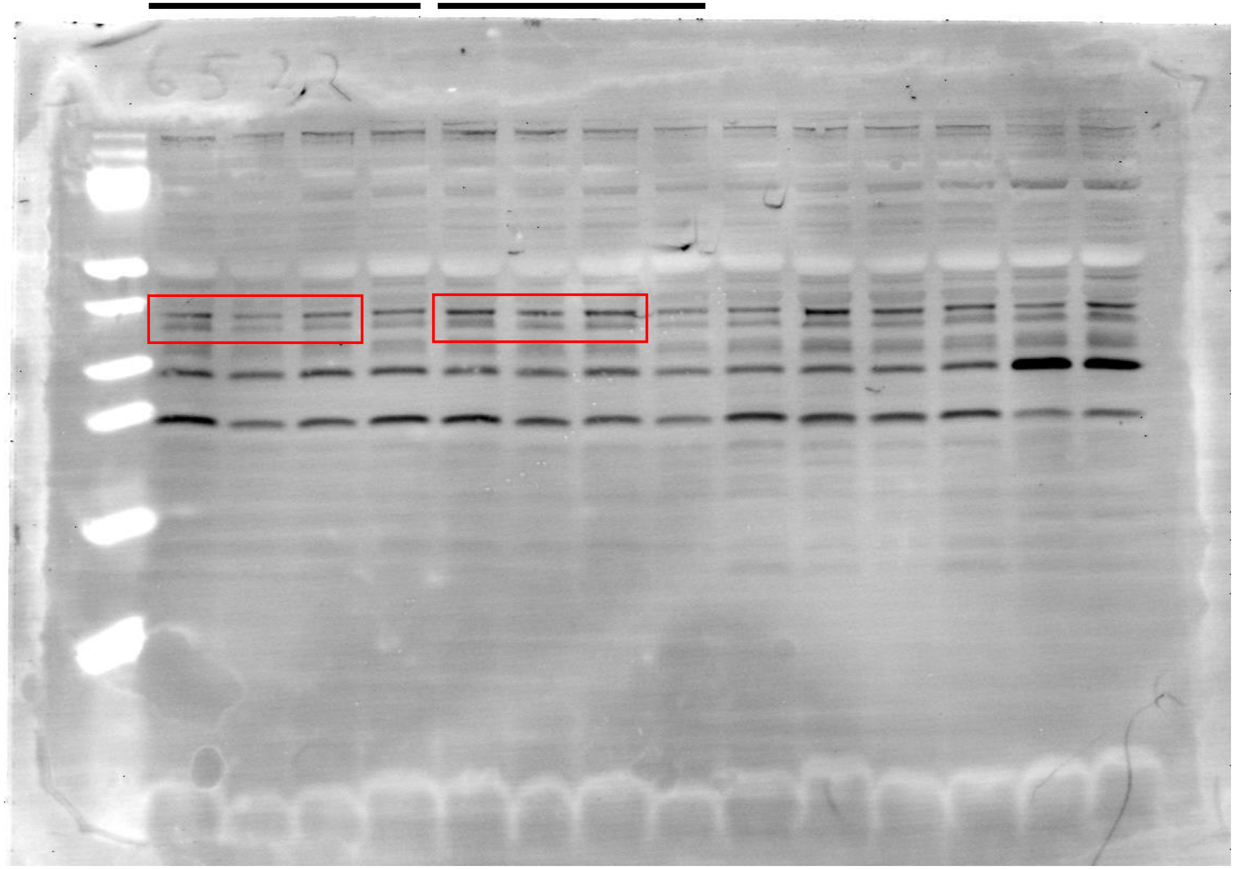

Sirt1

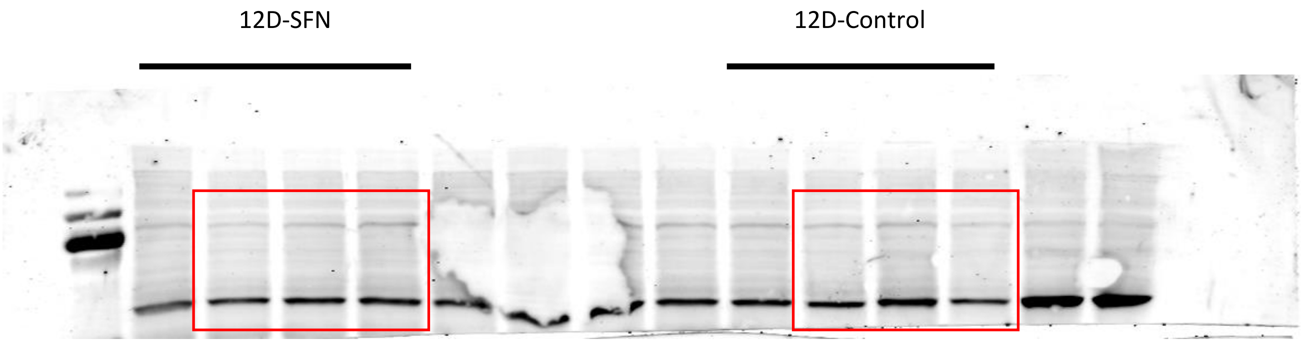

S6

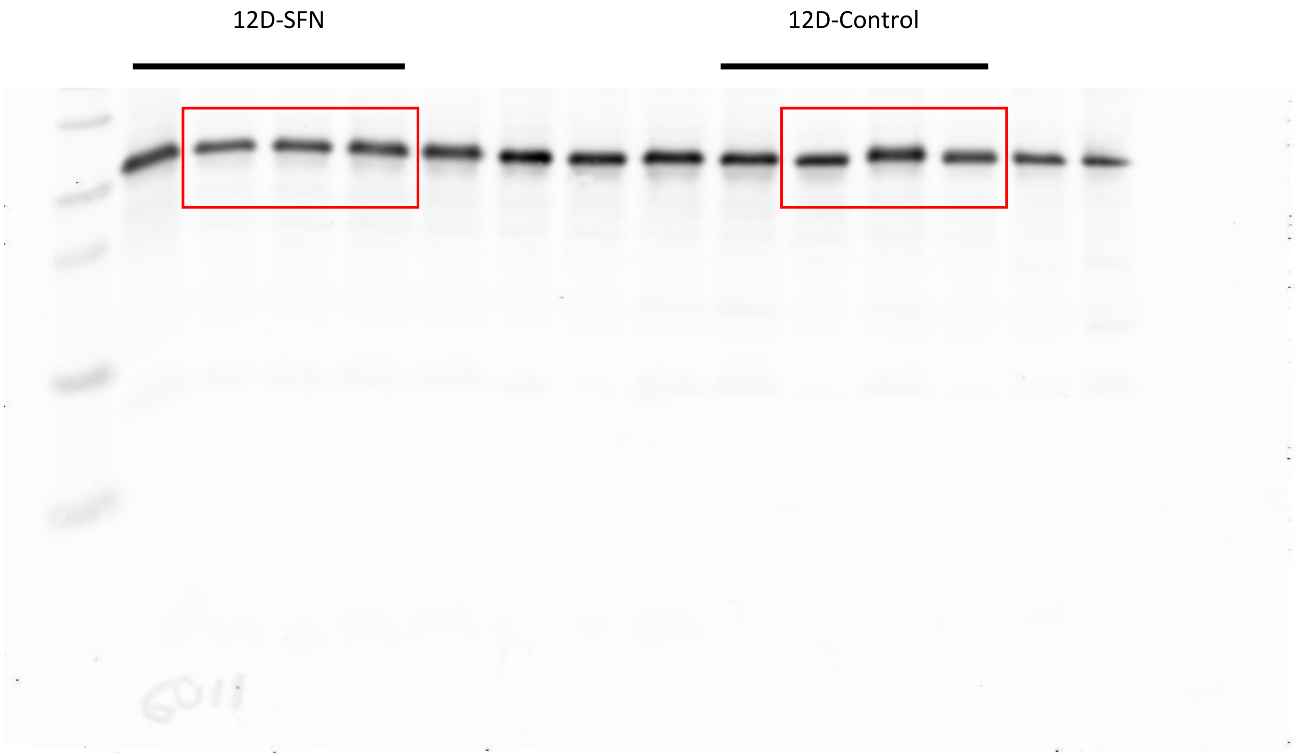

Pcbp1 – for Sirt1 and S6

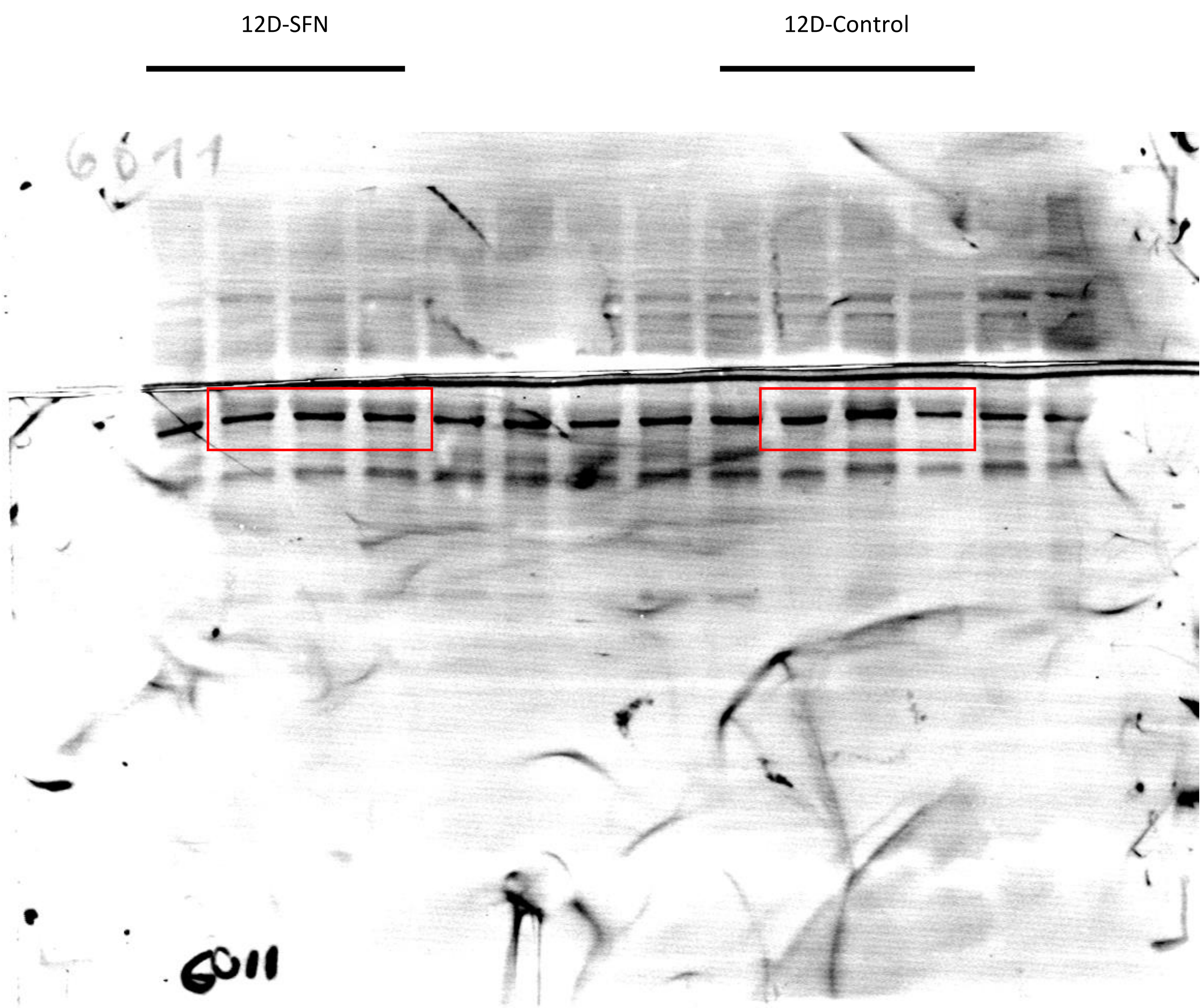

Parp1

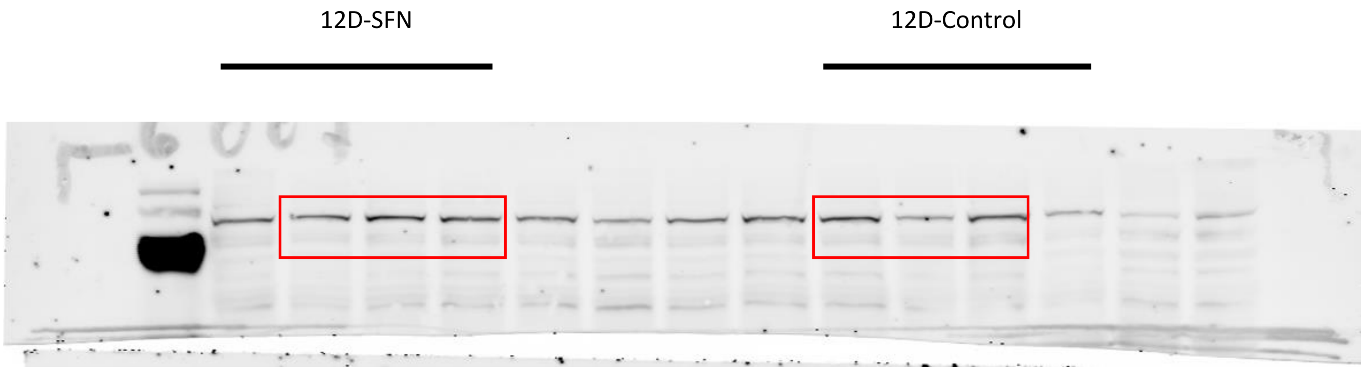

pS6

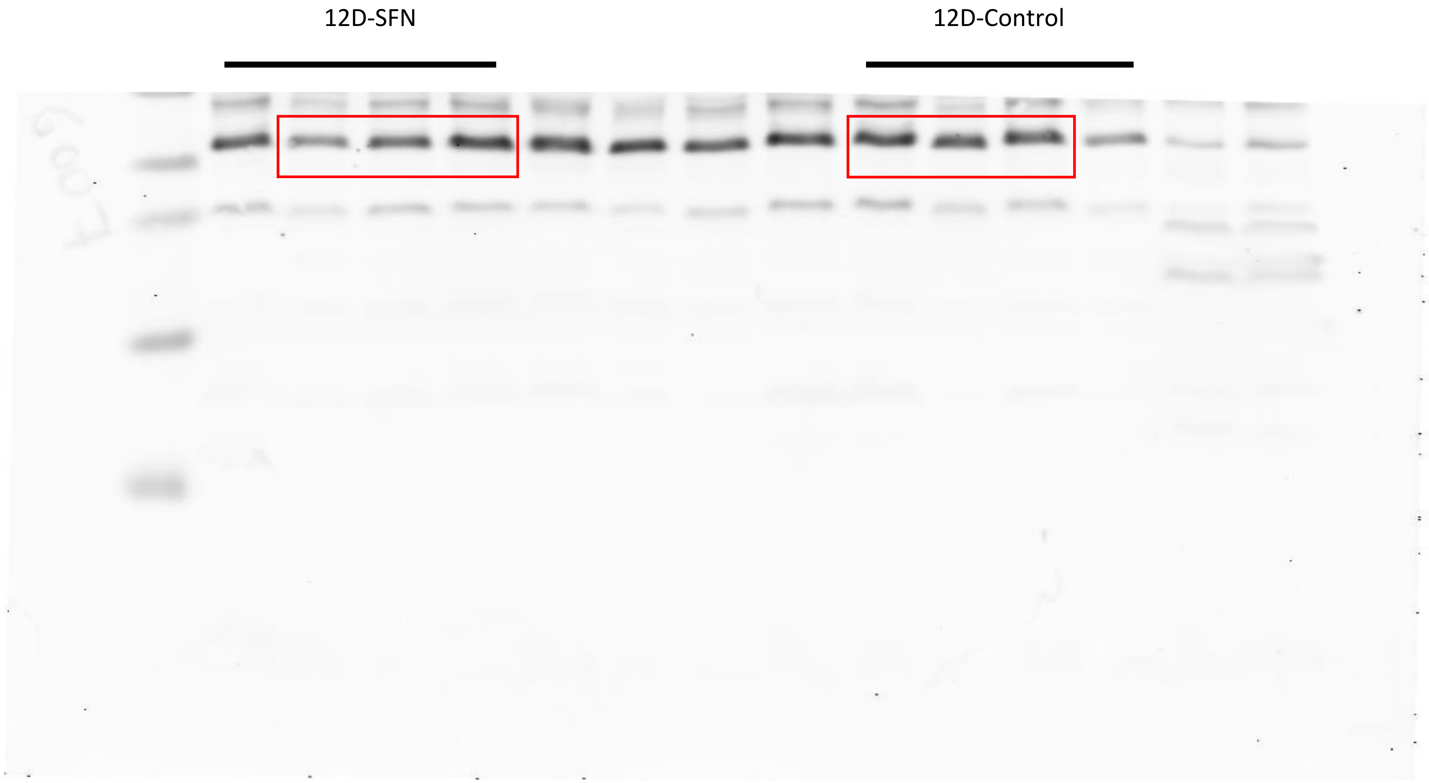

Pcbp1 – for Parp1, pS6

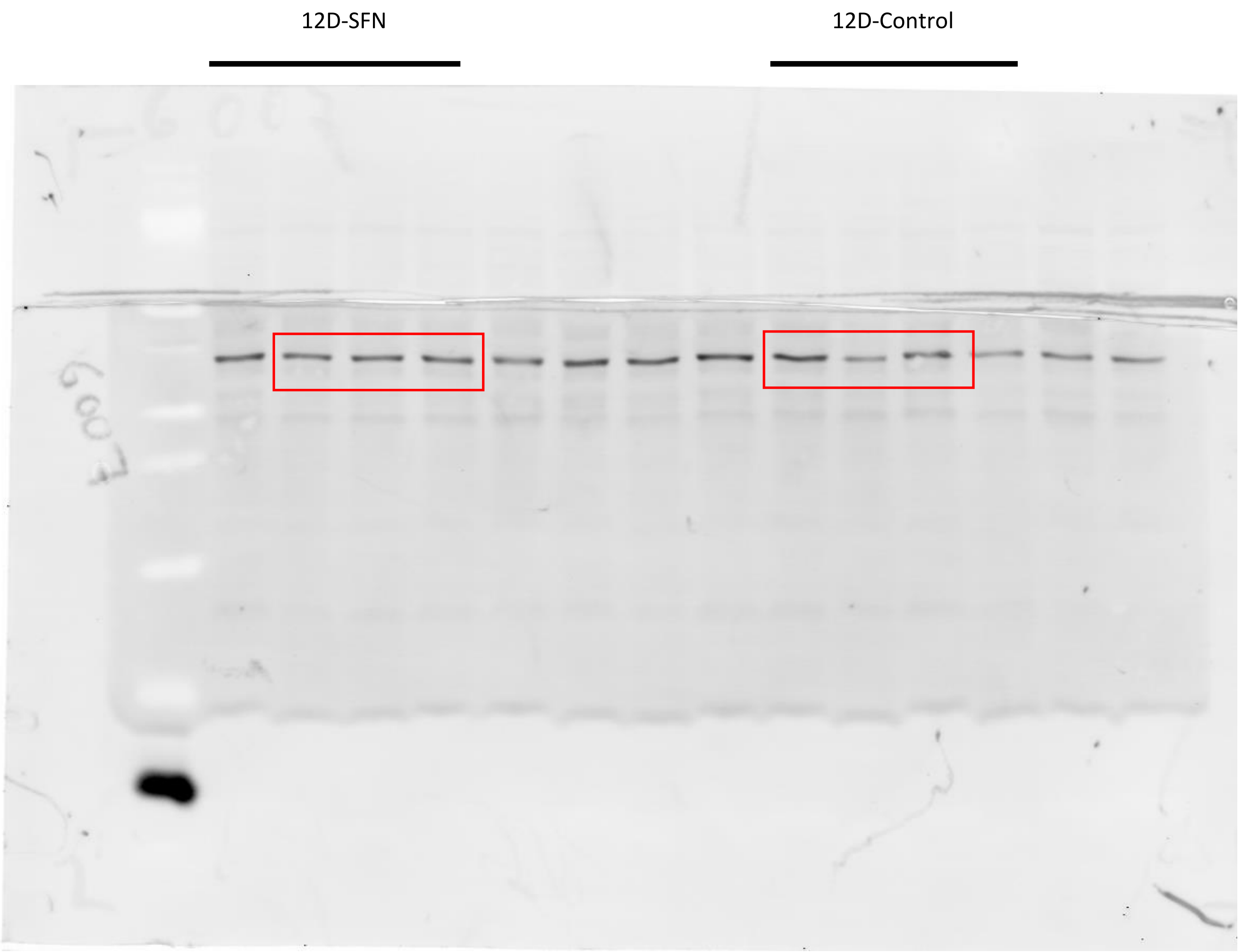

Supplement: Supplementary file 3 [file Data_Sheet_1.PDF]
